# Extended follow-up of invasive cervical cancer risk after quadrivalent HPV vaccination: nationwide, register based study

**DOI:** 10.1136/bmj-2025-087326

**Published:** 2026-02-25

**Authors:** Shiqiang Wu, Yunyang Deng, Tiia Lepp, Lina Schollin Ask, Pär Sparen, Mark Clements, Joakim Dillner, Jiayao Lei

**Affiliations:** 1 Department of Medical Epidemiology and Biostatistics, Karolinska Institutet, 171 77 Stockholm, Sweden; 2 Unit of Epidemiology, Institute of Environmental Medicine, Karolinska Institutet, Stockholm, Sweden; 3 Unit for Vaccination Programmes, Department of Public Health Analysis and Data Management, Public Health Agency of Sweden, Solna, Sweden; 4 Unit for Immunisation, Department of Communicable Disease Control and Health Protection, Public Health Agency of Sweden, Solna, Sweden; 5 Department of Women’s and Children’s Health, Karolinska Institutet, Stockholm, Sweden.; 6 Centre for Cervical Cancer Elimination, Department of Clinical Science, Intervention and Technology, Karolinska Institutet, Stockholm, Sweden

## Abstract

**Objectives:**

To evaluate the long term risk of invasive cervical cancer after receiving the quadrivalent human papillomavirus (HPV) vaccine, how risk varies by time since vaccination, and to assess the population level impact of HPV vaccination programmes.

**Design:**

Nationwide register based cohort study with up to 18 years of follow-up.

**Setting:**

Sweden, from 1 January 2006 to 31 December 2023.

**Participants:**

926​ 362 girls and women residing in Sweden between 2006 and 2023, born in 1985-88 (opportunistic cohort), 1989-92 (subsidised cohort), 1993-98 (catch-up cohort), or 1999-2001 (school based cohort), and with no previous HPV vaccination or diagnosis of invasive cervical cancer at the start of follow-up.

**Main outcome measures:**

Incidence rate ratios of invasive cervical cancer among vaccinated women versus unvaccinated women were estimated using Poisson regression, adjusting for age, calendar time, sociodemographic factors, and medical histories. Incidence rate ratios were further assessed by time since vaccination, stratified into three year intervals (eg, 1-3, 4-6 years), and by age at vaccination, as well as additional analyses by birth cohorts.

**Results:**

During follow-up, 365​ 502 (39.5%) girls and women received at least one dose of the quadrivalent HPV vaccine. 930 cases of invasive cervical cancer were identified, including 97 in vaccinated and 833 cases in unvaccinated individuals. Among participants vaccinated before 17 years, the overall fully adjusted incidence rate ratios compared with the unvaccinated group was 0.21 (95% confidence interval (CI) 0.13 to 0.32), with sustained protection for 13-15 years after vaccination (incidence rate ratio 0.23, 95% CI 0.11 to 0.46). For individuals vaccinated at 17 years or older, the overall fully adjusted incidence rate ratio was 0.63 (95% CI 0.49 to 0.81) compared with the unvaccinated group, with significant incidence reductions observed during years 10-12 (incidence rate ratio 0.54, 95% CI 0.33 to 0.86), and years 13-15 (incidence rate ratio 0.23, 95% CI 0.08 to 0.60) after vaccination. Compared with the opportunistic cohort, the school based cohort had a 72% (95% CI 11% to 91%) lower risk of cervical cancer after adjustment for covariates (incidence rate ratio 0.28, 95% CI 0.09 to 0.89).

**Conclusions:**

A significantly reduced risk of invasive cervical cancer following quadrivalent HPV vaccination persisted throughout long term follow-up, with no indication of waning protection. School based cohorts showed lower incidence of cervical cancer at the population level than the opportunistic cohort.

## Introduction

Persistent infection with high risk human papillomavirus (HPV) types is the primary cause of invasive cervical cancer.^1^ HPV vaccination offers a highly effective strategy to reduce the incidence of high grade cervical lesions and cervical cancer, by preventing infection with oncogenic HPV types.^2^ As a cornerstone of global efforts to eliminate cervical cancer, HPV vaccination has been incorporated into national immunisation programmes in about 150 countries.^3^ In Sweden, the quadrivalent HPV vaccine (which targets HPV types 6, 11, 16, and 18) was first approved for prophylactic use in late 2006.^2^ ^4^ Initially, vaccination was only available on an opportunistic, self-funded basis.^4^ In 2007, vaccination subsidised by the government was introduced for girls born between 1989 and 1992.^5^ This programme remained in place until 2012, when a school based vaccination programme was launched, offering the vaccine free of charge to girls born in 1999 or later.^5^ The school based programme was supported by a robust school health infrastructure, which likely contributed to high vaccine coverage and effective delivery.^5^ A parallel catch-up programme was also implemented for pupils born between 1993 and 1998.^5^ In 2015, the national schedule within the school based programme shifted from a three dose regimen to a two dose regimen.^5^ During this period, the quadrivalent vaccine was used almost exclusively until it was replaced in 2019 by the nonavalent vaccine, which additionally targets five other high risk HPV types (HPV 31, 33, 45, 52, and 58).^5^

Our previous nationwide cohort study in Sweden, which included up to 12 years of follow-up, demonstrated a significantly reduced risk of invasive cervical cancer following quadrivalent HPV vaccination.^6^ Studies from Denmark,^7^ England,^8^ ^9^ Scotland,^10^ and the Netherlands^11^ have also reported similar reductions in invasive cervical cancer from the HPV vaccination programmes. Although HPV vaccines have demonstrated high effectiveness against cervical cancer, evidence of sustained immunity is primarily immunological (eg, antibody titres),^12^ ^13^ rather than clinical (eg, disease rates). Evidence on the long term durability of vaccine protection against HPV related diseases (eg, invasive cervical cancer) is scarce, and it is also unclear how waning immunity, if present, may differ by age at vaccination. To fill these knowledge gaps, we extended follow-up on the risk of invasive cervical cancer after the quadrivalent HPV vaccine by an additional six years from our published study,^6^ and examined the risk reduction by time since vaccination. Furthermore, we assessed population level changes in incidence of invasive cervical cancer since the implementation of HPV vaccination programmes in Sweden.

## Methods

### Study design and study population

**Fig 1:**
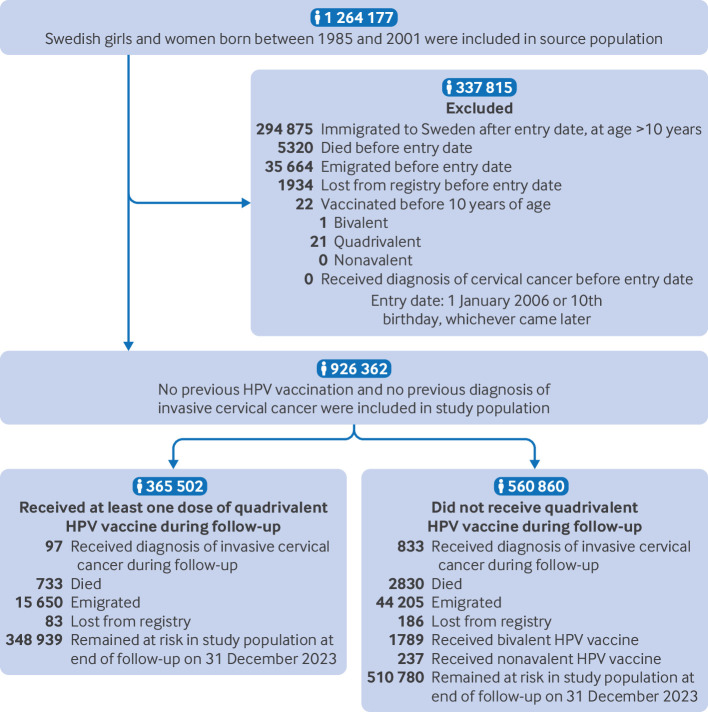
Population characteristics in study of invasive cervical cancer risk after quadrivalent HPV vaccination. HPV=human papillomavirus

Based on the Swedish Total Population Register,^14^ we conducted a register based cohort study that included all women born between 1985 and 2001 who were residents of Sweden between 2006 and 2023 (fig 1). All participants were followed from 1 January 2006, or from their 10th birthday, whichever came later. We excluded women who had died, had emigrated, were lost to follow-up, had received any types of HPV vaccinations, or had invasive cervical cancer before the start of the follow-up from the study population. Additionally, we excluded all girls and women who immigrated to Sweden after 2006 at an age older than 10 years, because HPV vaccination records from outside Sweden were not available. The eligible participants were followed until they received a diagnosis of invasive cervical cancer, died, emigrated, were lost to follow-up, received the bivalent or nonavalent HPV vaccination, or until 31 December 2023, whichever came first. Participants were followed up to a maximum age of 38 years, with those vaccinated before age 17 years followed up to a maximum age of 34 years. We used several Swedish national population and healthcare registers in this study and linked participant data at the individual level using the unique Swedish personal identity number, which is allocated to anyone intending to live in Sweden for more than one year.^14^

### HPV vaccination

Quadrivalent HPV vaccination status was treated as a time varying variable. Participants were considered to be vaccinated if they had received at least one dose of the vaccine. We retrieved vaccination records from the Swedish HPV Vaccination Register, National Vaccination Register, and Prescribed Drug Register. The Swedish HPV Vaccination Register is a voluntary national register that collected data on HPV vaccinations administered between 2006 and 2015.^6^ Since 2013, National Vaccination Register has provided virtually complete documentation of vaccinations from the national school based programme.^15^ ^16^ The Prescribed Drug Register contains records of all prescribed drugs dispensed in Sweden since 2005, including subsidised HPV vaccines given outside the organised programme.^17^ More details on the data collection of HPV vaccination initiatives are described in our earlier publications.^6^ ^18^

### Invasive cervical cancer

The outcome was defined as the first diagnosis of invasive cervical cancer during follow-up, with the use of code C53 in the ICD-10 (international classification of diseases, 10th revision). The date of diagnosis was retrieved from the Swedish Cancer Register, is a compulsory reporting system for all cancers founded in 1958 that covers the entire population and demonstrated high accuracy for classification.^19^ During the study period, all women aged 23 to 49 years were invited to participate in the nationwide, organised cervical cancer screening programme every three years.^20^

### Covariates

Covariates considered in the analysis included attained age, calendar year, county of residence, maternal country of birth, maternal history of high grade cervical lesions and non-cervical cancers, as well as the highest parental education and annual household income levels. All covariates, except attained age and calendar year, were measured as fixed variables before entry to the study. Data on covariates were retrieved from the Longitudinal Integration Database for Health Insurance and Labour Market Studies,^21^ the Swedish Cancer Register,^19^ and the Total Population Register.^14^

### Statistical analyses

The cumulative incidence of cervical cancer was estimated using the Kaplan-Meier method, stratified by HPV vaccination status and by birth cohorts, and plotted against age at follow-up. In the time varying setting, each participant’s follow-up time was divided into multiple shorter intervals based on attained age and, if applicable, the date of first HPV vaccination and time since vaccination (supplementary file, supplementary fig S1). Each interval reflected simultaneous changes in age (in one year intervals), calendar time (to capture temporal changes in background incidence and vaccination coverage; calculated as the sum of the birth year and attained age), and time since vaccination, where relevant. Person time was classified as unvaccinated or vaccinated according to the date of vaccination; the vaccinated person time was further stratified by age at vaccination and years since vaccination. Based on the median age at first sexual intercourse in Sweden (17 years),^22^ vaccinated individuals were initially categorised as vaccinated at age 10-16 years or ≥17 years. For more detailed stratification, we applied 15 years and 20 years as additional cut-off points, consistent with thresholds commonly used in studies of HPV vaccination.^6^ ^7^ We further categorised follow-up time among vaccinated individuals by time since vaccination in three year intervals (eg, 1-3 years, 4-6 years).

Incidence rate ratios and 95% confidence intervals (CIs) were estimated using Poisson regression models, comparing incidence rates between vaccinated and unvaccinated women. Vaccinated individuals were further stratified by age at vaccination and years since vaccination. Attained age served as the underlying timescale and was modelled as a natural spline term with 3 degrees of freedom. Fully adjusted models additionally controlled for calendar year (categorical, in one year bands), county of residence, maternal country of birth, parental highest education level, annual household income level, maternal history of high grade cervical lesions, and maternal history of non-cervical cancers. To handle missing data, we applied the missing indicator method by creating a separate “missing” category for covariates with incomplete values: 96.6% of participants had no missing data.

We also conducted an analysis by birth cohort to assess population level changes in cervical cancer incidence in relation to the implementation of HPV vaccination programmes. Participants were categorised into four birth cohorts based on their year of birth: 1985-88 (opportunistic cohort), 1989-92 (subsidised cohort), 1993-98 (catch-up cohort), and 1999-2001 (school based cohort). The opportunistic cohort was used as the reference group for comparisons. Poisson regression models were fitted to estimate incidence rate ratios for cervical cancer across the birth cohorts, with attained age as the underlying timescale. Models were adjusted for age as a spline term with 3 degrees of freedom, sociodemographic covariates, and maternal history of disease as in the primary analysis.

#### Sensitivity analyses

We imputed the missing covariate values using multiple imputation by chained equations with multinomial logistic models. We also stratified the comparison by birth cohort and attained age to assess whether our age adjustment adequately accounted for younger birth cohorts in the overall estimates. Additionally, we applied buffer periods of two, four, and six years between the date of vaccination and the start of case ascertainment to exclude possible HPV prevalent infections at the time of HPV vaccination.

All data management procedures were conducted using SAS version 9.4, and statistical analyses were performed with Stata version 18 (StataCorp). All tests were two sided, and significance was defined as P<0.05.

### Patient and public involvement

Patient and public involvement contributors were not formally involved in this research. However, our findings will be discussed and shared through updates of the National Swedish Guidelines for Cervical Cancer Prevention.^23^ The review process of updates includes feedback from both patients and professional organisations before the guidelines are finalised and implemented.

## Results

### Study population

In this study, we included 926​ 362 girls and women born between 1985 and 2001 and followed from 2006 to 2023, with a median follow-up of 18 years (interquartile range (IQR) 15.8-18.0 years; fig 1, table 1, and supplementary file, supplementary table S1). A total of 365 502 (39.5%) participants received at least one dose of the quadrivalent HPV vaccine during follow-up, of whom 74.2% initiated vaccination before age 17 years, and 76.5% received the full three dose schedule (supplementary file, supplementary table S2). Younger birth cohorts were generally characterised by higher HPV vaccine uptake and earlier age at vaccination (supplementary file, supplementary table S1). HPV vaccination was more common in individuals with Swedish born mothers, higher parental education or income, and with maternal medical conditions (supplementary file, supplementary table S1). During the study period, invasive cervical cancer was diagnosed in 833 participants in the unvaccinated group and 97 in the vaccinated group.

**Table 1:** Characteristics of the study population at baseline. Data are number (%) unless otherwise indicated

Characteristics	Unvaccinated	Vaccinated^*^	Vaccinated^* ^at age 10-14 years	Vaccinated^* ^at age 15-16 years	Vaccinated^*^ at age 17-19 years	Vaccinated^*^ at age ≥20 years
Total population	560 860 (60.5)	365 502 (39.5)	179 554 (49.1)	91 474 (25.0)	64 774 (17.7)	29 700 (8.1)
Median (IQR) age at first vaccination (years)	—	15.1 (13.0-17.1)	13.0 (12.0-14.0)	16.0 (15.5-16.5)	17.8 (17.4-18.4)	24.1 (22.3-25.7)
Birth cohort:						
1985-88	207 899 (93.2)	15 067 (6.8)	0	0	1520 (10.1)	13 547 (89.9)
1989-92	181 979 (72.9)	67 674 (27.1)	887 (1.3)	24 736 (36.6)	28 016 (41.4)	14 035 (20.7)
1993-98	137 257 (44.0)	175 080 (56.1)	72 766 (41.6)	65 786 (37.6)	34 495 (19.7)	2033 (1.2)
1999-2001	33 725 (23.9)	107 681 (76.2)	105 901 (98.4)	952 (0.9)	743 (0.7)	85 (0.1)
Mother’s country of birth:						
Sweden	425 872 (58.0)	308 292 (42.0)	148 959 (48.3)	79 715 (25.9)	55 067 (17.9)	24 551 (8.0)
Other country	116 841 (67.8)	55 449 (32.2)	29 722 (53.6)	11 347 (20.5)	9398 (17.0)	4982 (9.0)
Missing data†	18 147 (91.2)	1761 (8.9)	873 (49.6)	412 (23.4)	309 (17.6)	167 (9.5)
Highest parental education level:‡						
Low	36 636 (76.7)	11 110 (23.3)	5819 (52.4)	2096 (18.9)	2082 (18.7)	1113 (10.0)
Middle	276 064 (65.4)	145 854 (34.6)	70 699 (48.5)	36 505 (25.0)	27 741 (19.0)	10 909 (7.5)
High	228 953 (52.7)	205 719 (47.3)	101 035 (49.1)	52 376 (25.5)	34 708 (16.9)	17 600 (8.6)
Missing data†	19 207 (87.2)	2819 (12.8)	2001 (71.0)	497 (17.6)	243 (8.6)	78 (2.8)
Annual household income level:§						
Low	62 843 (66.8)	31 252 (33.2)	17 002 (54.4)	6847 (21.9)	5330 (17.1)	2073 (6.6)
Middle	219 622 (63.2)	128 064 (36.8)	64 329 (50.2)	31 195 (24.4)	23 141 (18.1)	9399 (7.3)
High	260 221 (56.1)	203 998 (43.9)	96 679 (47.4)	53 024 (26.0)	36 154 (17.7)	18 141 (8.9)
Missing data†	18 174 (89.2)	2188 (10.8)	1544 (70.6)	408 (18.7)	149 (6.8)	87 (4.0)
Maternal history of high grade cervical lesions:						
No	539 146 (60.5)	351 358 (39.5)	173 008 (49.2)	87 886 (25.0)	62 123 (17.7)	28 341 (8.1)
Yes	21 714 (60.6)	14 144 (39.4)	6546 (46.3)	3588 (25.4)	2651 (18.7)	1359 (9.6)
Maternal history of non-cervical cancers:						
No	545 893 (60.5)	356 450 (39.5)	175 602 (49.3)	89 216 (25.0)	62 993 (17.7)	28 639 (8.0)
Yes	14 967 (62.3)	9052 (37.7)	3952 (43.7)	2258 (24.9)	1781 (19.7)	1061 (11.7)

Percentages may not total 100 because of rounding. IQR=interquartile range.

* Received at least one dose of quadrivalent HPV vaccination.

† 96.6% of individuals did not have any missing data.

‡ The highest parental education level was classified as low if the parent had nine years or fewer of primary education, middle if the parent had 2-3 years of secondary schooling (similar to senior high school), and high if the parent had education after secondary schooling and above (equivalent to university studies).

§ Annual household income level was categorised into low, medium, and high based on the income level thirds (based on tertiles) of the population aged 20-65 years.

### Cumulative incidence of invasive cervical cancer

**Fig 2:**
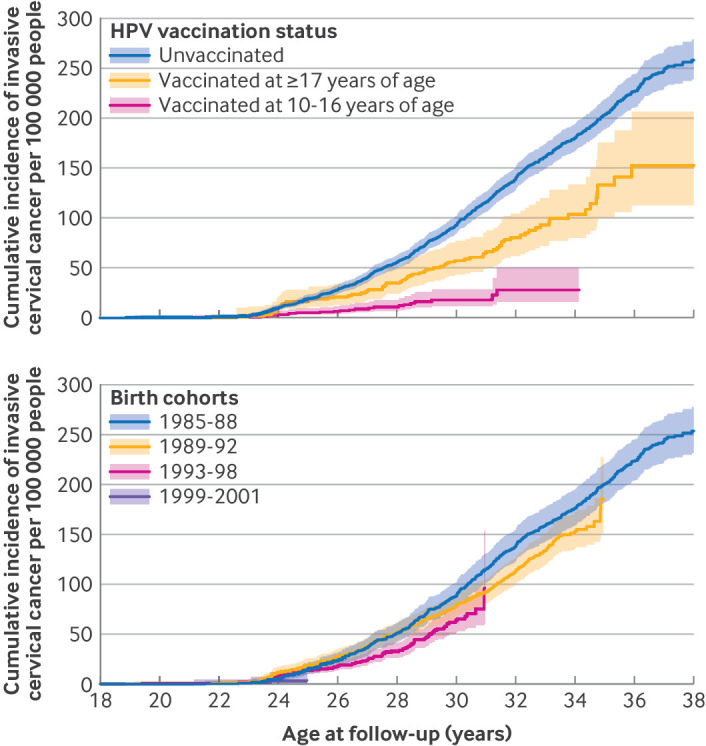
Cumulative incidence of invasive cervical cancer according to human papillomavirus (HPV; upper panel) vaccination status and birth cohorts (lower panel). Age at follow-up is truncated because no cases of cervical cancer were observed in participants under 18 years. In the lower panel, the steps at the end of follow-up are for events with very few individuals in the risk set

The cumulative incidence of invasive cervical cancer rose rapidly at 23 years of age across all groups (fig 2), aligning with the recommended age for initiating cervical cancer screening in Sweden.^20^ Overall, incidence was markedly lower among vaccinated women, particularly those vaccinated before 17 years (fig 2). By 34 years of age, the cumulative incidence was about 30, 100, and 180 per 100 000 persons among participants vaccinated before 17 years of age, those vaccinated after age 17 years, and unvaccinated women, respectively. The cumulative incidence in participants who had been vaccinated after 17 years and the unvaccinated group had increased to 150 and 260 per 100 000 persons, respectively, by age 38. Cumulative incidence of invasive cervical cancer decreased progressively across birth cohorts (fig 2). Women born in 1985-88 had the highest incidence of invasive cervical cancer, reaching around 250 cases per 100 000 by age 38. Subsequent cohorts of 1989-92 and 1993-98 showed progressively lower incidence. For cohort 1999-2001, the cumulative incidence was 4 per 100 000 during the available follow-up.

### Invasive cervical cancer by HPV vaccination status

The overall fully adjusted incidence rate ratio comparing women who received at least one dose of the quadrivalent HPV vaccine with unvaccinated women was 0.44 (95% CI, 0.35 to 0.55; table 2). When stratified by age at vaccination, those vaccinated before age 17 years had a substantially lower risk of cervical cancer, with an incidence rate ratio of 0.21 (95% CI 0.13 to 0.32), compared to 0.63 (0.49 to 0.81) among those vaccinated at 17 years or older. Among women vaccinated before age 17, the incidence rate ratios were comparable between participants vaccinated at ages 10-14 years (incidence rate ratio 0.20, 95% CI 0.10 to 0.40) and 15-16 years (0.20, 0.12 to 0.35). For women who were vaccinated at age 17-19 years and after 20 years, the incidence rate ratios were 0.54 (95% CI 0.39 to 0.76) and 0.76 (0.54 to 1.08), respectively.

**Table 2:** Incidence rate ratios of invasive cervical cancer in cohorts stratified by HPV vaccination status and age of vaccination initiation

HPV vaccination status	Person years	No of cases of invasive cervical cancer	Age adjusted incidence rate ratio (95% CI)*	Fully adjusted incidence rate ratio (95% CI)†	Relative risk reduction (%; 95% CI)‡
Unvaccinated	10 787 209	833	Reference	Reference	Reference
Vaccinated	4 439 152	97	0.41 (0.33 to 0.51)	0.44 (0.35 to 0.55)	56 (45 to 65)
Age at first vaccination (years):					
10-16	3 322 508	24	0.21 (0.14 to 0.31)	0.21 (0.13 to 0.32)	79 (68 to 87)
10-14	2 136 320	9	0.21 (0.11 to 0.41)	0.20 (0.10 to 0.40)	80 (60 to 90)
15-16	1 186 188	15	0.20 (0.12 to 0.34)	0.20 (0.12 to 0.35)	80 (65 to 88)
≥17	1 116 645	73	0.60 (0.47 to 0.76)	0.63 (0.49 to 0.81)	37 (19 to 51)
17-19	817 287	39	0.54 (0.39 to 0.74)	0.54 (0.39 to 0.76)	46 (24 to 61)
≥20	299 357	34	0.68 (0.48 to 0.96)	0.76 (0.54 to 1.08)	24 (−8 to 46)

HPV=human papillomavirus; CI=confidence interval.

* Adjusted for age as a spline term with 3 degrees of freedom.

† Adjusted for age as a spline term with 3 degrees of freedom, calendar year, county of residence in the year before study entry, mother’s country of birth, highest parental education level, annual household income level, and maternal history of high grade cervical lesions and noncervical cancers.

‡ Calculated as 1 minus the fully adjusted incidence rate ratio.

### Invasive cervical cancer by time since vaccination

**Fig 3:**
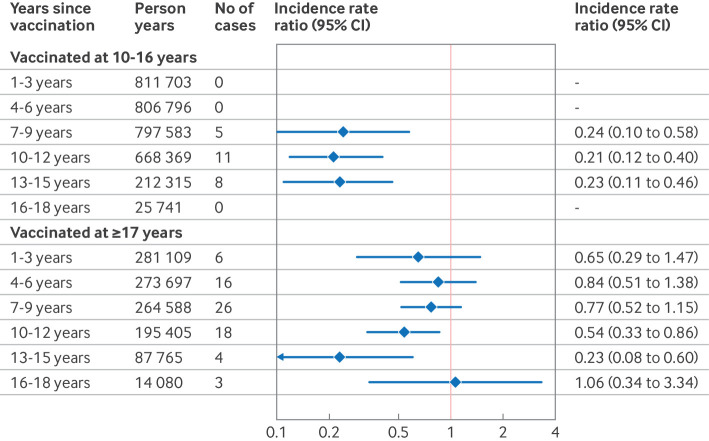
Incidence rate ratios of invasive cervical cancer by age at HPV vaccination and years since the first dose of HPV vaccination. Combined estimates for 13-18 years since vaccination: vaccinated at 10-16 years, incidence rate ratios 0.20 (0.10 to 0.40); vaccinated at ≥17, incidence rate ratio 0.34 (0.16 to 0.72). Incidence rate ratios were adjusted for age as a spline term with 3 degrees of freedom, calendar year, county of residence in the year before study entry, mother’s country of birth, highest parental education level, annual household income level, and maternal history of high grade cervical lesions and non-cervical cancers. CI=confidence interval

Among girls who received at least one dose of HPV vaccination between the ages of 10 and 16 years, we observed 811 703 person years within one to three years after vaccination, which progressively decreased over time to 25 741 person years during 16-18 years after vaccination (fig 3). During the 7-9, 10-12, and 13-15 years since vaccination, the fully adjusted incidence rate ratios were 0.24 (95% CI 0.10 to 0.58), 0.21 (0.12 to 0.40), and 0.23 (0.11 to 0.46), respectively. Among women who initiated vaccination after age 17, the fully adjusted incidence rate ratios were 0.65 (95% CI 0.29 to 1.47), 0.84 (0.51 to 1.38), 0.77 (0.52 to 1.15), 0.54 (0.33 to 0.86), and 0.23 (0.08 to 0.60) for the intervals of 1-3, 4-6, 7-9, 10-12, and 13-15 years since vaccination, respectively. For women vaccinated either before or after 17 years of age, we had insufficient statistical power to estimate vaccine effectiveness 16 to 18 years after vaccination owing to the limited number of person years and cases in this interval. The combined estimates for ages 13-18 years were 0.20 (95% CI 0.10 to 0.40) for those vaccinated at ages 10-16 years and 0.34 (0.16 to 0.72) for those vaccinated at age 17 years or older (fig 3).

### Invasive cervical cancer by birth cohorts

Among women born between 1985 and 1988, the coverage of the vaccine was 6.8%, and the median age at first dose was 25.3 years (IQR 21.8-26.5). Among the subsidised cohort (1989-92), the catch-up cohort (1993-98), and school based cohort (1999-2001), the median age at vaccination was 17.4 years (IQR 16.6-19.4), 15.4 years (14.4-16.7) and 12.2 years (11.6-12.8), respectively. Compared with the opportunistic cohort, the overall fully adjusted incidence rate ratios were 0.84 (95% CI 0.72 to 0.97) for the subsidised cohort, 0.67 (95% CI 0.54 to 0.84) for the catch-up cohort, and 0.28 (95% CI 0.09 to 0.89) for the school based cohort (table 3).

**Table 3:** Incidence rate ratios of invasive cervical cancer by birth cohorts eligible for various HPV vaccination programmes

Birth cohort (vaccination programme)	Vaccination coverage (%)§	No of person years	No of cases of invasive cervical cancer	Age adjusted incidence rate ratio (95% CI)*	Fully adjusted incidence rate ratio (95% CI)†	Relative risk reduction (%; 95% CI)‡
1985-88 (opportunistic cohort)	6.8	3 780 956	493	Reference	Reference	Reference
1989-92 (subsidised cohort)	27.1	4 301 826	312	0.85 (0.73 to 0.99)	0.84 (0.72 to 0.97)	16 (3 to 28)
1993-98 (catch-up cohort)	56.1	5 270 602	122	0.67 (0.54 to 0.84)	0.67 (0.54 to 0.84)	33 (16 to 46)
1999-2001 (school based cohort)	76.2	1 872 978	3	0.27 (0.09 to 0.87)	0.28 (0.09 to 0.89)	72 (11 to 91)

HPV=human papillomavirus; CI=confidence interval.

^§^ Received at least one dose of quadrivalent HPV vaccination.

* Adjusted for age as a spline term with 3 degrees of freedom.

† Adjusted for age as a spline term with 3 degrees of freedom, county of residence in the year before study entry, mother’s country of birth, highest parental education level, annual household income level, and maternal history of high grade cervical lesions and noncervical cancers.

‡ Calculated as 1 minus the fully adjusted incidence rate ratio.

### Sensitivity analyses

Estimated incidence rate ratios after multiple imputations were consistent with those from the main analysis (supplementary file, supplementary table S3). Stratifying the comparisons of birth cohorts by attained age gave a fully adjusted incidence rate ratio for the school based cohort of 0.20 (95% CI 0.05 to 0.84) among women aged 22-24 years compared with that of the opportunistic cohort in the corresponding age groups (supplementary file, supplementary table S4). The incidence rate ratios (overall and age specific) of invasive cervical cancer after HPV vaccination remained consistent across buffer periods of two, four, and six years (supplementary file, supplementary table S5). Among participants vaccinated before age 17 years, the introduction of a buffer period had minimal impact on the estimated incidence rate ratios. Among women who initiated vaccination between the ages of 17 and 19 years, the point estimates declined to some extent with longer buffer periods, with an incidence rate ratio of 0.45 (95% CI 0.31 to 0.66) when a six year buffer period was applied. However, no significant protection was observed among women vaccinated after 20 years across any buffer periods, although the point estimates indicated lower incidence rate ratios.

## Discussion

### Principal findings

This nationwide cohort study with up to 18 years of follow-up found that risk of invasive cervical cancer remained substantially reduced after quadrivalent HPV vaccination. This risk reduction was observed regardless of age at vaccination initiation, with no indication of waning or attenuation over time. To our knowledge, this is the first study to assess how the risk reduction varies by time since HPV vaccination based on the longest follow-up. In addition, benefitting from Sweden’s substantial efforts to increase HPV vaccine uptake, we observed a substantial reduction in cervical cancer incidence at the population level, particularly among birth cohorts covered by the school based HPV vaccination programme. These findings reinforce current strategies that prioritise vaccination at younger ages, particularly through school based programmes targeting girls at a very young age.

Among women vaccinated before age 17 years, the absence of cancer cases in the first six years and the 16-18 years after vaccination initiation highlights the necessity of sufficiently long follow-up when evaluating routine vaccination programmes, to ensure reliable estimates of the risk reduction. No significant protection was observed during years 1-9 after vaccination among women vaccinated at age 17 or older. This is in line with an earlier UK modelling study that projected most of the benefit of HPV vaccination would only become apparent after a decade of follow-up.^24^ However, we observed significant risk reduction during years 10-12 and 13-15 after vaccination, suggesting a delayed observation of reduction in incidence at the population level among women who are vaccinated at older ages. This delayed risk reduction may reflect earlier HPV exposure at the time of vaccination, as the vaccine is prophylactic and does not treat existing infections.^25^ Given the long progression from HPV infection to invasive cervical cancer,^26^ the risk reduction during the early years since vaccination may have been confounded by pre-existing HPV infections. These findings underscore the importance of extended follow-up when evaluating HPV vaccine effectiveness among women vaccinated at older ages and highlight the need to account for earlier HPV infection (eg, by using longer buffer periods in future analyses).

### Comparison with other studies

Previous studies from Sweden,^6^ Denmark,^7^ England,^8^ ^9^ Scotland,^10^ and the Netherlands^11^ have demonstrated that HPV vaccination can effectively reduce the incidence of cervical cancer at the population level. Our findings further reinforce this evidence by showing sustained risk reduction for invasive cervical cancer for 15 years or longer after vaccination, particularly among women vaccinated before age 17 years, with no indication of waning protection. This finding is supported by long term clinical and immunological studies.^27^ ^28^ A large prospective cohort study in India found that the quadrivalent HPV vaccine provided high protection against persistent type 16 HPV and type 18 HPV infections for up to 15 years.^27^ Similarly, studies based on data from an international, randomised, double blind phase 3 trial of the quadrivalent HPV vaccine (FUTURE II) in Finland showed that protective antibody titres remained detectable up to 12 years after vaccination,^13^ and demonstrated excellent vaccine efficacy against cancers associated with HPV with up to 11 years of follow-up.^29^ In addition, our findings highlight the importance of school based HPV vaccination programmes to eliminate cervical cancer, consistent with previous evidence from England which demonstrated around 84% lower incidence of cervical cancer for cohorts vaccinated through routine vaccination.^9^

The risk reduction for individuals who were vaccinated after 20 years of age seems to be relatively limited, even when applying buffer periods of two to six years, consistent with findings from a registry based study in Denmark.^7^ However, immunological studies have shown that HPV vaccination in adulthood can still elicit seropositivity and good humoral immune responses.^12^ ^30^ Furthermore, our previous research demonstrated that women vaccinated between the ages of 21 and 35 years had a significantly reduced risk of high grade cervical lesions after receiving two or three doses of the quadrivalent HPV vaccine compared with unvaccinated individuals.^18^

### Strengths and limitations of this study

The first strength of this study is that using high quality, nationwide Swedish health and population registers generated a large cohort with up to 18 years of follow-up. This setting provided sufficient statistical power to examine the risk reduction of invasive cervical cancer across multiple periods since vaccination. Secondly, our analyses adjusted for a wide range of potential confounding factors, including sociodemographic and parental characteristics. We also carefully applied multiple timescales, such as attained age and calendar year, into our models. Thirdly, we compared cervical cancer incidence across birth cohorts vaccinated through various HPV vaccination strategies, which provided insights into the impact of national immunisation strategies at a population level. Such an approach enables efficient data retrieval from national registers, facilitating long term follow-up and future surveillance of the effects of vaccine initiatives, especially when a strong herd effect is present.

Some limitations exist: a small proportion of vaccinated women may have been misclassified as unvaccinated, which could bias our estimates towards the null. Between 2006 and 2015, approximately 8% of HPV vaccine doses recorded in the Swedish HPV Vaccination Register were anonymised because of a lack of informed consent.^6^ In addition, Sweden launched a new vaccination initiative in 2021 targeting women born between 1994 and 1999, but the vaccinations administered through this programme were not captured in our database.^31^ However, since we adjusted for calendar year and the follow-up time for this cohort was limited to only one to two years, the impact on our estimates is likely to be limited.^18^ Secondly, we were unable to assess the HPV type distribution among incident cases, which limits an evaluation of the type specific risk reduction for cancer. However, using overall invasive cervical cancer as the outcome captures the real world impact of vaccination at the population level. Next, because our study is observational, residual confounding may be present. A healthy volunteer bias is also possible, as women who chose to receive the HPV vaccination may have been generally healthier than those who did not.^6^ Moreover, lifestyle factors like smoking and sexual activity could not be adjusted for, as they are not recorded in national registers. To partially account for these biases, we used parental education and household income as proxy indicators.^32^ Lastly, owing to the substantially lower incidence of cervical cancer among vaccinated women, we had limited statistical power to evaluate the dose specific risk reduction by each year since vaccination. However, comparable risk reduction for high grade cervical lesions across different doses received has been reported, particularly among women who are vaccinated at younger ages.^18^ This limitation highlights the need for continued long term follow-up on the effect of HPV vaccination.

### Conclusion and policy implications

In our register based study of individuals born between 1985 and 2001 in Sweden, with up to 18 years of follow-up after HPV vaccination, we found sustained risk reduction of invasive cervical cancer after quadrivalent HPV vaccine. No indication of waning protection was observed among the vaccinated population. These findings further support global strategies aimed at cervical cancer elimination through high vaccine coverage, particularly in younger populations, and emphasise the critical role of routine immunisation programmes.

**What is already known on this topic**

Human papillomavirus (HPV) vaccination significantly reduces invasive cervical cancer risk among Swedish girls and women aged 10-30 years, with the greatest benefit observed in those vaccinated at a younger age
Since the introduction of the quadrivalent HPV vaccine in 2006, data on long term risk of invasive cervical cancer after HPV vaccination remain limited
Long term follow-up studies are critical to assess the durability of risk reduction after HPV vaccination

**What this study adds**

With an additional six years of follow-up until 2023, our data confirm sustained cervical cancer risk reduction after quadrivalent HPV vaccination in Sweden
A consistently low incidence of invasive cervical cancer was observed up to 18 years after vaccination, with no indication of waning protection regardless of age at vaccination initiation
A population level decline in invasive cervical cancer incidence was demonstrated among cohorts vaccinated through national initiatives, highlighting the important role of organised vaccination in reducing the burden of cervical cancer

## Data Availability

The register data used in this study are not publicly available due to legal and ethical restrictions under European Union and Swedish data protection legislation, including the General Data Protection Regulation (GDPR). Access to individual level data from Swedish national health and population registers is strictly regulated by law and can only be granted after ethical review and approval by the appropriate authorities in Sweden. More detailed information can be requested from Swedish National Board of Health and Welfare (registerservice@socialstyrelsen.se) and Statistics Sweden (scb@scb.se). The analysis code is openly available at https://doi.org/10.5281/zenodo.17571204.

## Web extra

Extra material supplied by authors

Web appendix: Supplementary file

## Notes

Contributors: SW was responsible for data curation, formal analysis, investigation, methodology, validation, visualisation, writing, reviewing, and editing the manuscript. YD, MC, LSA, TL, JD, and PS were involved in investigation, supervision, validation, and reviewing, and editing of the manuscript. JL was responsible for conceptualisation, funding acquisition, project administration, supervision, methodology, validation, and reviewing, and editing of the manuscript. All authors read and approved the submitted version. SW and JL are the guarantors. ChatGPT 4.1 (OpenAI) was used solely to improve the clarity and language of the manuscript. All content was reviewed and approved by the authors. The corresponding author attests that all listed authors meet authorship criteria and that no others meeting the criteria have been omitted.

Transparency: The lead authors (SW and JL) affirm that the manuscript is an honest, accurate, and transparent account of the study being reported; that no important aspects of the study have been omitted; and that any discrepancies from the study as originally planned (and, if relevant, registered) have been explained.

Dissemination to participants and related patient and public communities: The work will be disseminated to policymakers, professional societies, and relevant patient and public communities through multiple channels, including press releases, targeted social media outreach, presentations at stakeholder meetings, and academic conferences.

Provenance and peer review: Not commissioned; externally peer reviewed.
