# Supplementary material for: Extended follow-up of invasive cervical cancer risk after quadrivalent HPV vaccination: nationwide, register based study
Source: BMJ. 2026 Feb 25;392:e087326. doi: 10.1136/bmj-2025-087326 (PMC12934514; doi:10.1136/bmj-2025-087326)
Supplement: Supplementary file 1 — Web appendix: Supplementary file [file wush087326.ww.pdf]

Table of Contents

Figure S1. Schematic illustration of time-varying exposure by attained age and years since vaccination 2

Table S1. The characteristics of birth cohorts 3

Table S2. Number (column %) of vaccinated study participants by total doses of quadrivalent HPV vaccination received during the follow-up 4

Table S3. Incidence rate ratios of invasive cervical cancer and HPV vaccination status using missing indicator and multiple imputation 5

Table S4. Incidence rate ratios (IRRs) with 95% confidence intervals (CIs) of invasive cervical cancer by birth cohorts and attained age 6

Table S5. Incidence rate ratios of invasive cervical cancer in relation to HPV vaccination status with and without a buffer period of two to six years 7

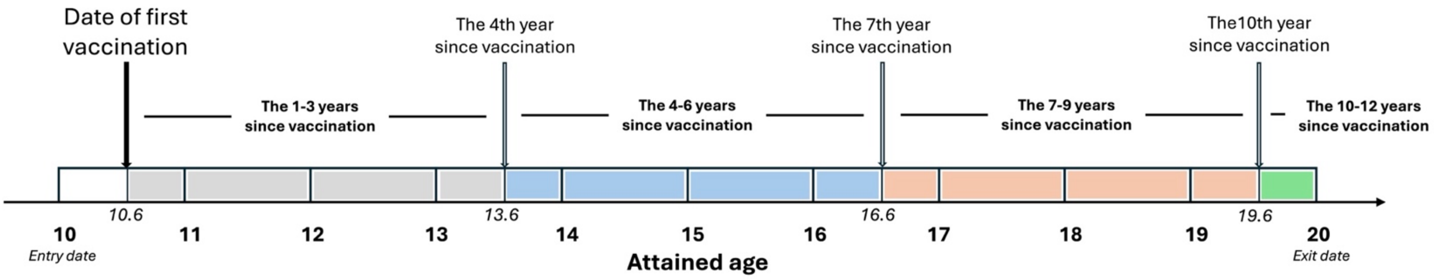

Figure S1. Schematic illustration of time-varying exposure by attained age and years since vaccination

Table S1. The characteristics of birth cohorts

	**1985-1988** Opportunistic	**1989-1992** Subsidized	**1993-1998** Catch-up	**1999-2001** School-based	**Total**
**Median follow-up years (IQR)**	18.0 (18.0 to 18.0)	18.0 (18.0 to 18.0)	18.0 (16.4 to 18.0)	13.5 (12.6 to 14.2)	18.0 (15.8 to 18.0)
**Median Vaccination Age (IQR)**	25.3 (21.8 to 26.5)	17.4 (16.6 to 18.4)	15.4 (14.4-16.7)	12.2 (11.6 to 12.8)	15.1 (13.0 to 17.1)
**No. of Vaccinated Individual (%)** [a]	15,067 (6.8)	67,674 (27.1)	175,080 (56.1)	107,681 (76.2)	365,502 (39.5)
**Mother's country of birth — no. (vaccinated rate, %)** [b]					
Sweden	12,891 (7.3)	59,890 (29.5)	150,150 (61.2)	85,361 (78.8)	308,292 (42.0)
Other country	2,093 (5.5)	7,473 (17.8)	24,171 (39.9)	21,712 (68.7)	55,449 (32.2)
Missing data	83 (1.2)	311 (6.2)	759 (11.9)	608 (39.9)	1,761 (8.9)
**Highest parental education level — no. (vaccinated rate, %)** [b]					
Low	432 (2.9)	1,411 (10.9)	4,840 (35.6)	4,427 (71.1)	11,110 (23.3)
Middle	4,435 (4.3)	24,303 (20.3)	73,881 (52.3)	43,235 (75.4)	145,854 (34.6)
High	10,159 (10.4)	41,878 (37.2)	95,236 (63.8)	58,446 (78.2)	205,719 (47.3)
Missing data	41 (0.7)	82 (1.8)	1,123 (13.6)	1,573 (51.9)	2,819 (12.8)
**Annual household income level — no. (vaccinated rate, %)** [b]					
Low	772 (3.8)	3,959 (17.0)	14,423 (43.7)	12,098 (69.6)	31,252 (33.2)
Middle	3,842 (5.0)	19,833 (21.2)	63,807 (51.8)	40,582 (74.6)	128,064 (36.8)
High	10,397 (8.7)	43,791 (34.1)	95,989 (64.5)	53,821 (80.2)	203,998 (43.9)
Missing data	56 (0.9)	91 (2.2)	861 (11.6)	1,180 (46.8)	2,188 (10.8)
**Maternal history of high-grade cervical lesions — no. (vaccinated rate, %)** [b]					
No	14,303 (6.7)	64,607 (27.0)	168,499 (55.8)	103,949 (76.0)	351,358 (39.5)
Yes	764 (7.5)	3,067 (29.4)	6,581 (62.4)	3,732 (79.9)	14,144 (39.4)
**Maternal history of noncervical cancers — no. (vaccinated rate, %)** [b]					
No	14,376 (6.7)	65,475 (27.0)	171,164 (55.9)	105,435 (76.1)	356,450 (39.5)
Yes	691 (8.4)	2,199 (32.9)	3,916 (62.9)	2,246 (79.0)	9,052 (37.7)

IQR, interquartile range.

a. Received at least one dose of quadrivalent HPV vaccination.
b. Percentage of individuals vaccinated with at least one dose of the quadrivalent HPV vaccine within each subgroup.

Table S2. Number (column %) of vaccinated study participants by total doses of quadrivalent HPV vaccination received during the follow-up

		Age at first vaccination				Birth cohorts			
	**Total**	**10-14**	**15-16**	**17-19**	**20+**	**1985-1988**	**1989-1992**	**1993-1998**	**1999-2001**
**One**	39,377 (10.8)	18,135 (10.1)	7,157 (7.8)	7,904 (12.2)	6,181 (20.8)	2,594 (17.2)	5,103 (7.5)	17,267 (9.9)	14,413 (13.4)
**Two**	46,370 (12.7)	18,709 (10.4)	10,896 (11.9)	10,005 (15.5)	6,760 (22.8)	2,983 (19.8)	7,638 (11.3)	24,726 (14.1)	11,023 (10.2)
**Three**	279,755 (76.5)	142,710 (79.5)	73,421 (80.3)	46,865 (72.4)	16,759 (56.4)	9,490 (63.0)	54,933 (81.2)	133,087 (76.0)	82,245 (76.4)
**Total**	365,502	179,554	91,474	64,774	29,700	15,067	67,674	175,080	107,681

Table S3. Incidence rate ratios of invasive cervical cancer and HPV vaccination status using missing indicator and multiple imputation

HPV Vaccination Status	Fully adjusted incidence rate ratio [a] (95% CI)	
	Main Analysis (missing indicator)	Sensitivity Analysis (multiple imputation) [b]
**Unvaccinated**	Reference	Reference
**Vaccinated**	0.44 (0.35 to 0.55)	0.45 (0.36 to 0.57)
Age at first vaccination		
10-16 yr	0.21 (0.13 to 0.32)	0.21 (0.14 to 0.33)
10-14 yr	0.20 (0.10 to 0.40)	0.21 (0.11 to 0.42)
15-16 yr	0.20 (0.12 to 0.35)	0.21 (0.12 to 0.36)
17 + yr	0.63 (0.49 to 0.81)	0.65 (0.50 to 0.83)
17-19 yr	0.54 (0.39 to 0.76)	0.56 (0.40 to 0.78)
20 + yr	0.76 (0.54 to 1.08)	0.78 (0.55 to 1.11)

HPV, human papillomavirus; CI, confidence interval.

a. Adjusted for age as a spline term with 3 degrees of freedom, calendar year, county of residence in the year before study entry, mother's country of birth, highest parental education level, annual household income level, and maternal history of high-grade cervical lesions and noncervical cancers.
b. In total, 96.6% of individuals in our cohort had complete covariate data, corresponding to an overall missingness of 3.4%, with 1.8-2.4% for any single covariate. Missingness was confined to four variables: county of residence, mother's country of birth, highest parental education, and annual household income. Missing values were handled using multiple imputation by chained equations (MICE) with multinomial logistic models, including invasive cervical cancer incidence, HPV vaccination status, age and calendar year at entry, maternal history of high-grade cervical lesions, and noncervical cancers as predictors. Ten imputed datasets (m=10) were generated with the augment option to improve convergence, and estimates from the imputed datasets were combined using Rubin's rules.

Table S4. Incidence rate ratios (IRRs) with 95% confidence intervals (CIs) of invasive cervical cancer by birth cohorts and attained age

	Attained age			
**Birth Cohort**	**18-21**	**22-24**	**25-30**	**31-34**
**1985-1988 (Opportunistic cohorts)**	Reference	Reference	Reference	Reference
**1989-1992 (Subsidized cohorts)**	1.25 (0.11 to 13.79)	1.15 (0.73 to 1.82)	0.72 (0.59 to 0.89)	0.98 (0.76 to 1.25)
**1993-1998 (Catch-up cohorts)**	1.94 (0.22 to 17.37)	0.86 (0.54 to 1.37)	0.51 (0.39 to 0.66)	-[a]
**1999-2001 (School-based cohorts)**	1.04 (0.06 to 16.86)	0.20 (0.05 to 0.84)	-[a]	

IRRs adjusted for county of residence in the year before study entry, mother's country of birth, highest parental education level, annual household income level, and maternal history of high-grade cervical lesions and noncervical cancers.

a. The cohort did not reach this age during the follow-up.

Table S5. Incidence rate ratios of invasive cervical cancer in relation to HPV vaccination status with and without a buffer period of two to six years

Buffer Period	HPV Vaccination Status	No. of Cases of Invasive Cervical Cancer	Age-adjusted incidence rate ratio [a] (95% CI)	Fully adjusted incidence rate ratio [b] (95% CI)
No buffer period	Unvaccinated	833	Reference	Reference
	Vaccinated	97	0.41 (0.33 to 0.51)	0.44 (0.35 to 0.55)
	10-16 yr	24	0.21 (0.14 to 0.31)	0.21 (0.13 to 0.32)
	17 + yr	73	0.60 (0.47 to 0.76)	0.63 (0.49 to 0.81)
	17-19 yr	39	0.54 (0.39 to 0.74)	0.54 (0.39 to 0.76)
	20 + yr	34	0.68 (0.48 to 0.96)	0.76 (0.54 to 1.08)
2-year	Unvaccinated	838	Reference	Reference
	Vaccinated	92	0.40 (0.32 to 0.50)	0.42 (0.33 to 0.54)
	10-16 yr	24	0.21 (0.14 to 0.31)	0.21 (0.13 to 0.32)
	17 + yr	68	0.58 (0.45 to 0.75)	0.61 (0.48 to 0.79)
	17-19 yr	39	0.54 (0.39 to 0.74)	0.55 (0.39 to 0.76)
	20 + yr	29	0.65 (0.45 to 0.94)	0.73 (0.50 to 1.07)
4-year	Unvaccinated	842	Reference	Reference
	Vaccinated	88	0.40 (0.32 to 0.50)	0.43 (0.34 to 0.54)
	10-16 yr	24	0.21 (0.14 to 0.31)	0.21 (0.14 to 0.32)
	17 + yr	64	0.60 (0.46 to 0.77)	0.63 (0.49 to 0.83)
	17-19 yr	37	0.52 (0.38 to 0.73)	0.53 (0.38 to 0.75)
	20 + yr	27	0.74 (0.51 to 1.09)	0.85 (0.57 to 1.25)
6-year	Unvaccinated	855	Reference	Reference
	Vaccinated	75	0.37 (0.29 to 0.47)	0.39 (0.30 to 0.51)
	10-16 yr	24	0.21 (0.14 to 0.32)	0.22 (0.14 to 0.33)
	17 + yr	51	0.56 (0.42 to 0.74)	0.59 (0.44 to 0.79)
	17-19 yr	29	0.44 (0.30 to 0.64)	0.45 (0.31 to 0.66)
	20 + yr	22	0.83 (0.55 to 1.28)	0.95 (0.62 to 1.46)

HPV, human papillomavirus; CI, confidence interval.

a. Adjusted for age as a spline term with 3 degrees of freedom.
b. Adjusted for age as a spline term with 3 degrees of freedom, calendar year, county of residence in the year before study entry, mother’s country of birth, highest parental education level, annual household income level, and maternal history of high-grade cervical lesions and noncervical cancers.
